# Feasibility of the trial procedures for a randomized controlled trial of a community-based peer-led wheelchair training program for older adults

**DOI:** 10.1186/s40814-017-0158-3

**Published:** 2017-07-17

**Authors:** Krista L. Best, William C. Miller, François Routhier, Janice J. Eng

**Affiliations:** 1 Department of Rehabilitation, Université Laval, Quebec City, QC Canada; 2 Center for Interdisciplinary Research in Rehabilitation and Social Integration, Centre integré de santé et de services sociaux de la Capitale-Nationale, Institut de réadaptation en déficience physique de Québec, Quebec City, QC Canada; 3 The Department of Occupational Sciences and Occupational Therapy, Faculty of Medicine, University of British Columbia, Vancouver, BC Canada; 4 The Department of Physical Therapy, Faculty of Medicine, University of British Columbia, Vancouver, BC Canada; 5 The Rehabilitation Research Program, Vancouver Coastal Research Institute, GF Strong Rehabilitation Centre, Vancouver, BC Canada; 6 Rehabilitation Research Lab, UBC Department of Occupational Science & Occupational Therapy, Vancouver, BC Canada

**Keywords:** Manual wheelchair, Older adults, Peer training, Rehabilitation, Self-efficacy

## Abstract

**Background:**

A novel peer-led manual wheelchair (MWC) training program may support the training needs of older adults, but establishing program feasibility is a pragmatic first step. The purpose of this study was to evaluate the feasibility of a peer-led Wheelchair training Self-Efficacy Enhanced for Use (WheelSeeU) program.

**Methods:**

Forty MWC users (mean age 65 years) were randomly assigned to the experimental (WheelSeeU) or control group. Feasibility indicators of process, resources, management, and safety were collected throughout the study.

**Results:**

The consent rate was 49%. Participant retention rate was 90% post-intervention and 87.5% at follow-up (6 months). All participants reported perceived benefits from WheelSeeU. Participants and trainers adhered to the study protocol (>90%), and fidelity of the WheelSeeU intervention was attained (>90%). There were no adverse events.

**Conclusions:**

WheelSeeU is an innovative and feasible approach for providing MWC training to older adults that is accessible beyond initial rehabilitation without increased clinician burden. With minor modifications, it is feasible that WheelSeeU can be administered to older adults living in the community.

**Trial registration:**

NCT01838135.

## Background

Manual wheelchair (MWC) mobility is of particular concern among older adults (i.e., ≥65 years) who comprise more than 50% of the wheelchair user demographic [1]. Optimizing wheelchair skills can positively influence wheelchair use [2] and may reduce risk of accidents [3] and the mobility dependence of more than 50% of older wheelchair users [4]. Although 5 h or less of manual wheelchair skills training effectively improves wheelchair skills capacity when administered by a healthcare professional in rehabilitation or community contexts [5–8], older adults receive little to no wheelchair skills training [9, 10]. Results of a recent survey from a rehabilitation centre highlight this issue, as only 55% of adults (~58 years of age) reported receiving wheelchair skills training before discharge. The remaining 45% did not know of arrangements to receive any training after discharge [11].

Wheelchair skills training facilitated by health care professionals during initial rehabilitation may be inhibited by clinician-perceived barriers of limited time and resources, clinicians’ skills and knowledge of wheelchair training, competing priorities during rehabilitation stays, and readiness for wheelchair use among patients [12, 13]. Moreover, many older adults procure wheelchairs independently in the community (e.g., directly from vendors, secondhand, or online) without clinical guidance and likely do not receive any training at all. While community-based training programs may address issues related to scheduling, readiness, and procurement of wheelchairs without the advice of a clinician, reliance solely on clinicians to implement such programs may pose additional burden and inferred healthcare costs.

The concept of wheelchair use self-efficacy, defined as an individual’s belief in his or her ability to overcome challenging situations when using a wheelchair [14], is a powerful mediator of use and participation in older manual wheelchair users [15, 16]. Self-efficacy, important for adult learning, is enhanced when individuals learn from people they perceive to be similar to themselves and who are managing similar situations [17]. Therefore, implementing peers to facilitate interventions may provide an effective strategy for modeling wheelchair use. A peer-led wheelchair training program showed preliminary effectiveness for improving wheelchair use self-efficacy and wheelchair skills in adult MWC users 19 years of age and older [18].

Additional benefits of a peer-led program may include increased social interactions [19] or improved cost-effectiveness when compared to professional-led interventions [20]. Moreover, peer-led wheelchair programs may provide an avenue for fostering a needed continuum of wheelchair skills training that starts during initial rehabilitation and continues after community reintegration [18]. However, before proceeding with larger effectiveness trials, it is critical to first evaluate the feasibility of peer-led wheelchair training for older adults [21]. Establishing feasibility will confirm key issues related to a study’s process and resources. Additionally, findings from feasibility studies can be used to optimize the design of subsequent effectiveness trials and judge appropriateness of proceeding with larger trials [21].

Considering the need for community-based training for older adult MWC users and the importance of establishing feasibility, the purpose of this study was to evaluate the feasibility of a peer-led wheelchair training program called “Wheelchair training Self-Efficacy Enhanced for Use (WheelSeeU)” [22].

The specific study objectives were to evaluate the feasibility according to the following indicators: (1) *process issues*: recruitment rate, consent rate, retention rate, and perceived benefit; (2) *resource issues*: participant adherence, trainer adherence, burden of data collection, and translation of study materials; (3) *management issues*: participant processing time, combining data in English and French, study protocol administration, and fidelity of WheelSeeU intervention; and (4) *safety*: safety during the intervention and data collection (modified from [21]).

## Methods

A full description of the methods has been previously published [22]. A brief description is detailed below. Minor modifications have been made to the study protocol since the original publication, including the following: (1) Feasibility indicators for treatment response and variation of outcomes were not reported in this feasibility study and will be reported in a subsequent paper; (2) the eligibility criteria was revised from ≥55 to ≥50 years of age to increase the potential participant pool; (3) success on one the feasibility indicators for process (i.e., recruitment rate) was modified from >90% acceptance to >20% acceptance to accurately reflect the reality of recruitment in rehabilitation research; and (4) the training protocols for the intervention and the control groups were modified slightly to accommodate the need for individualized training if one of the participants missed a training session.

### Design

A two-site randomized controlled trial (RCT) was completed with community-dwelling MWC users in Québec City, QC, Canada, and Vancouver, BC, Canada.

### Participants

#### Inclusion criteria

- ≥50 years of age
- Lived in the community
- Could self-propel a MWC an average of 1 h per day
- Had self-proclaimed wheelchair mobility goals
- Was cognitively able to engage in the WheelSeeU program (Modified Mini-Mental Status Exam (MMSE) score of ≥24) [23]

#### Exclusion criteria

- Unable to complete study questionnaires in English or French
- Had anticipated health conditions or procedures that contraindicate training (e.g., surgery that may impair physical activity)
- Had degenerative conditions that were expected to progress quickly (e.g., amyotrophic lateral sclerosis)
- Were receiving or planning to receive wheelchair mobility training during the study period

### Recruitment

Recruitment took place between October 2013 and March 2016 using the following recruitment strategies: database of existing wheelchair users, clinicians, and vendors described the study to inpatients and outpatients at a local rehabilitation centre; posters and presentations given to special interest groups in the community; mail-outs to potential participants as identified by hospital records; social media (i.e., Facebook page); and word-of-mouth. The protocol for this study was approved by the Research Ethics Boards at the University of British Columbia, Institut de réadaptation en déficience physique de Québec, and Vancouver Coastal Health in Vancouver. All study participants provided informed consent.

### Randomization and concealment

The randomization procedure was designed by an offsite statistician and consisted of stratification by site using block sizes of four or six (to allow for randomization of pairs of participants). When two participants were enrolled, the Tester completed baseline data collection and entered data into a secure database. The Site Coordinator then contacted the statistician to obtain group assignment for the pair of participants within 48 h. The Site Coordinator forwarded participant contact information to the appropriate group trainer (i.e., intervention or control group), who contacted both participants to schedule six training sessions. Therefore, randomization was concealed from the Tester at all time points.

### Intervention and control groups

The WheelSeeU intervention consisted of six (~weekly), 1.5-h wheelchair training sessions delivered by a peer-trainer and a support-trainer to two wheelchair users. Peer-trainers who were ≥45 years of age, who had at least 5 years of experience using a wheelchair (with intermediate to advanced wheelchair skills), and support-trainers who had at least 5 years of clinical experience were recruited and trained in a 2-day workshop (~15 h) led by a study investigator (KB).

WheelSeeU incorporated tasks known to challenge wheelchair use self-efficacy (e.g., navigating a MWC in the community, completing activities in a MWC, managing social situations in a MWC [14]), but each session was individualized according to participant-defined goals. After introductions, the peer-trainer gave a brief presentation outlining expectations of WheelSeeU. The peer-trainer explained the SMART goal framework [24], then worked with participants at the beginning of WheelSeeU to define MWC mobility or participation-oriented goals. Goals were recorded, progression was monitored, and participants were encouraged to set new goals as their skills progressed. The peer-trainer and support-trainer worked together to break the goals into smaller task objectives (and to identify potential barriers), which helped them to form the training plan for each session. The task objectives were built into practice, as well as methods for overcoming barriers. The peer-trainer provided verbal instruction, demonstration, and feedback to participants (with help from the support-trainer when necessary). The peer-trainer debriefed the participants at the end of each session to recap goals and discussed how to integrate new skills into practice in the community and at home (i.e., provided homework). The peer-trainer took note of the participants’ homework, which formed the initial discussion for the next session. The support-trainer provided spotting during the practice of skills and intervened immediately if there was unsafe wheeling that could lead to tips or falls. The future intent of WheelSeeU is that it will be administered by a peer-trainer, with spotting provided by caregivers, family members, volunteers, or clinician assistants. However, given that this is a feasibility study of a novel intervention, it was prudent to ensure participant safety through the inclusion of a clinician support-trainer.

Participants in the control group received six (~weekly) 1.5-h didactic sessions of information about using a wheelchair in the community (iWheel) led by a health care professional. The iWheel protocol, created specifically for this study, was designed to control for attention without providing any wheelchair skills training (available at http://millerresearch.osot.ubc.ca). Each session started with an “icebreaker” activity (e.g., “MWC Jeopardy,” “Describe your dream vacation”), and then, information (and embedded questions) were presented on six topics: (1) accessible places in the community (e.g., How do you determine whether a place is accessible prior to going there?); (2) transportation (e.g., Describe your experiences using public transportation/ trains/ ferries/ airplanes?); (3) MWC set-up and maintenance (e.g., What regular maintenance do you do on your MWC?); (4) using computers (e.g., How do you use the internet?); (5) pain and fatigue management (e.g., What do you do to manage pain/ fatigue?); and (6) Physical activity and nutrition (e.g., What physical activities do you take part in using your MWC?). Pre-formatted questions were integrated to reduce the likelihood of spontaneous discussions about wheelchair skills among participants. A health care or research professional with at least 2 years of experience in rehabilitation research completed 6 h of iWheel training facilitated by a study investigator (KB). The iWheel trainer was instructed not to give any MWC skills training or advice on how to use a MWC in a better way.

### Outcomes to assess feasibility

Feasibility indicators for process, resources, management, and safety parameters were measured during study administration and at study end.

#### Process indicators

*Recruitment rate*—The recruitment rate was defined as the number of participants recruited per month. This information was recorded in the study log.

*Consent rate—*The consent rate was calculated by dividing the number of individuals who met inclusion criteria, by the number who consented to participate in the study. A research coordinator recorded the reasons why eligible individuals were not interested in participating in the study log.

*Retention rate*—The retention rate was calculated by dividing the number of participants, who completed data collection at T2 and T3, by the number of participants who completed data collection at T1.

*Perceived benefit—*Participant perceived benefit of the intervention was assessed using a study-specific survey that asked participants if they felt WheelSeeU had any benefits towards using a MWC.

### Resource indicators

*Participant adherence*—Adherence to the study was assessed by tracking the total number of WheelSeeU and iWheel sessions attended by participants. The trainers recorded participant adherence in a study log.

*Trainer recruitment and adherence*—The ability to recruit and maintain a peer-trainer (i.e., trainer adherence) was assessed by tracking the total number of WheelSeeU sessions attended by the peer-trainer.

*Participant and tester burden—*Burden was measured by the amount of time it took to administer study outcomes at T1, T2, and T3. The tester recorded this data at each time point.

Data collection commenced in October 2013 and was completed in October 2016.

Descriptive variables

Sociodemographic and personal information (i.e., age, sex, marital status, highest level of education, cognition, primary diagnosis related to MWC use, length of time using the MWC, propulsion method, depression (Hospital Anxiety and Depression Scale (HADS) [25]), and social support (Interpersonal Support Evaluation List (ISEL) [26]) were collected at baseline.

Clinical outcome measures:

The proposed primary outcome for the main trial, wheelchair skills capacity, was assessed objectively using the Wheelchair Skills Test (WST) [27] at baseline (T1), post-intervention (T2), and 3-month follow-up (T3). Secondary clinical outcomes were also collected for the main trial at the three time points and included subjective wheelchair skills capacity and performance and safety (Wheelchair Skills Test Questionnaire (WST-Q) [28]), wheelchair use self-efficacy (Wheelchair Use Confidence Scale [29]), life-space mobility (Life-Space Assessment (LSA) [30]), satisfaction with participation (Wheelchair Outcome Measure (WhOM) [31]), quality of life (Late-life Function and Disability Index (LLFDI) [32]), and health utility (Health Utility Index (HUI) [33]). However, only preliminary results of the primary clinical outcome (objective wheelchair skills capacity) are reported in this paper. Clinical outcomes have been described previously [22], and results will be presented in a separate manuscript.

*Ability to translate/complete study in English and French*—Based on monthly meetings among study investigators and research coordinators, the ability to translate and complete the study protocol was gauged through subjective evaluation (i.e., yes or no).

### Management indicators

*Participant processing time*—Participant processing time was defined as the total number of days from initial contact to study enrolment. Details on processing time were recorded in the study log by the research coordinator.

*Ability to combine data—*The ability to combine the data was gauged through subjective evaluation of study investigators.

*Administration of study protocol*—To ensure the protocol was administered as intended (i.e., training was completed with pairs of participants by a peer-trainer and a support-trainer), study protocol administration was guided by a protocol checklist. The checklist was monitored and recorded by the support-trainer and control group trainer.

*Fidelity*—Intervention fidelity, defined as adherent and competent delivery of the intervention, was evaluated using a study-specific WheelSeeU Administrator Rating Form that outlined important details and components of the WheelSeeU intervention to be completed by the peer-trainer and the support-trainer in a checklist (e.g., support-trainer demonstrated proper application of the spotter strap, peer-trainer helped to develop new goals and reviewed existing goals with participants). A research coordinator at both sites completed the WheelSeeU Administrator Rating Form randomly, at least one time per participant pair during the WheelSeeU intervention.

### Safety

*Intervention—*Intervention safety was measured by the number of adverse events that occurred during the WheelSeeU intervention (e.g., tips, falls, cuts, abrasions, blisters). The support-trainer was responsible for documenting any adverse events.

*Data collection—*Safety during data collection was assessed by the number of adverse events that occurred during testing procedures. The tester was responsible for recoding any adverse events that occurred during data collection.

### Analysis

Descriptive statistics (mean, standard deviation (SD), counts (percentage)) were used to summarize continuous and categorical data as appropriate. Feasibility indicators for process, resources, management, and treatment were treated as binary (i.e., successful, unsuccessful). “Successful” indicated that the protocol is sufficiently robust to move forward with a large RCT with only small or no adaptation to the protocol required, while “unsuccessful” indicated a need for changes to the protocol before proceeding.

Sample size was calculated a priori using variability data (mean (SD)) of the WST from three randomized controlled trials [22]. According to Campbell et al. [34], a sample size of 40 is large enough to represent the target population and to evaluate feasibility indicators.

## Results

**Fig. 1:**
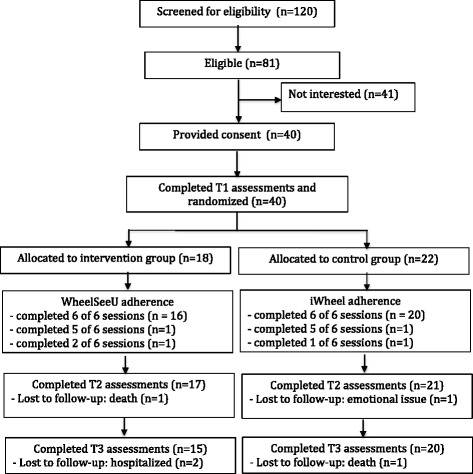
Flow of participants through the WheelSeeU study

The flow of participants through the WheelSeeU study is described in Fig. 1.

Participants had a mean (SD) age of 64.5 (8.0) years, predominantly male (60%), with primary diagnoses of amputation (28%), spinal cord injury (20%), and other conditions (e.g., multiple sclerosis, stroke, Parkinson’s disease, post-polio) (52%), and had a mean (SD) of 7 (11.3) years of previous experience using a manual wheelchair. Table 1 provides sample characteristics and baseline demographic information. The groups were reasonably well balanced after randomization, but the intervention group reported a higher level of depression and the control group had more years of previous wheelchair experience.

**Table 1:** Demographic, wheelchair-related, and clinical variables at baseline

Participant characteristics	WheelSeeU (*n* = 18)	Control (*n* = 22)
Demographic and personal information		
Age, year, mean (SD); range	66.2 (7.0); 54–83	63.1 (8.7); 50–84
Sex, no. (%)		
Male	7 (39)	17 (77)
Marital status, no. (%)		
Married or common law	10 (56)	11 (50)
Education, no. (%)		
College or university	15 (83)	16 (73)
Income CAD, no. (%)		
<15,000	1 (5)	5 (23)
15,000–50,000	8 (44)	8 (36)
Primary language, no. (%)		
English	4 (22)	10 (45)
Primary diagnosis, no. (%)		
Spinal cord injury	3 (17)	5 (23)
Amputation	3 (17)	8 (36)
Other (MS, stroke, Parkinson’s, post-polio)	12 (67)	9 (41)
Wheelchair-related variables		
Previous MWC use, year, mean (SD); range	4.3 (5.5); 0–22	9.0 (14.0); 0–45
Use in current MWC, year, mean (SD); range	2.8 (5.3); 0–22	1.3 (2.0); 0–10
Use MWC daily		
Yes	14 (78)	17 (77)
Propulsion method, no. (%)		
2 hands only	16 (89)	17 (77)
Hours per day spent in MWC, no. (%)		
>8	6 (33)	8 (36)
5–8	4 (22)	3 (13)
WC-related accident in the past year, no. (%)		
Yes	2 (11)	1 (5)
Clinical variables at baseline		
MMSE, mean (SD); range [max score 30]	28.2 (1.3); 26–30	28.8 (0.9); 27–30
ISEL, mean (SD); range [max score 18]	10.5 (5.3); 3–18	15.6 (3.6); 7–18
HADS anxiety, mean (SD); range [max score 21]	6.5 (6.4); 0–16	4.0 (2.4); 0–7
HADS depression, mean (SD); range [max score 21]	7.3 (3.4); 4–12	2.0 (1.3); 1–4

*MMSE* The Mini-Mental State Examination, *ISEL* Interpersonal Support Evaluation List, *HADS* Hospital Anxiety and Depression Scales

### Summary of clinical outcomes (baseline)

Summary statistics (i.e., mean (SD)) of primary and secondary clinical outcomes for the intervention and control groups at baseline are presented in Table 2. After randomization, the intervention group had slightly lower mean scores on all clinical outcomes compared to the control group.

**Table 2:** Baseline summaries of primary and secondary clinical outcomes

Clinical outcomes	Intervention group (*n* = 18) Mean (SD)	Control group (*n* = 22) Mean (SD)
WST (max score 100)		
Objective wheelchair skills capacity	66.0 (13.3)	71.6 (11.4)
WST-Q (max score 100)		
Subjective wheelchair skills capacity	67.0 (15.7)	76.1 (10.0)
Subjective wheelchair skills performance	45.5 (20.7)	56.3 (17.3)
WheelCon (max score 100)		
Wheelchair use self-efficacy	65.9 (22.7)	79.6 (13.8)
WhOM (max score 100)		
Satisfaction with participation	55.3 (24.8) (*n* = 17)	62.9 (27.2) (*n* = 21)
LSA (max score 120)		
Life-space mobility	34.9 (21.0)	44.7 (22.9)
LLFDI (max score 100)		
Participation frequency	49.5 (9.1)	53.5 (11.7)
LLFDI (max score 100)		
Instrumental role	34.3 (9.9)	44.0 (12.3)

*WST* Wheelchair Skills Test, *WST-Q* Wheelchair Skills Test Questionnaire, *WheelCon* Wheelchair Use Confidence Scale, *WhOM* Wheelchair Outcome Measure, *LSA* Life-Space Assessment, *LLFDI* Late-Life Function and Disability Index

### Feasibility indicators

Success was achieved on 10 of 13 feasibility indicators. Definitions of feasibility indicators and a priori parameters for success are defined in Table 3.

**Table 3:** Description of feasibility indicators, parameters for success, results, and suggested modifications

Feasibility indicator	Outcome measure	Parameter for success	Results	Feasible	Suggested modifications
Process (*n* = 4)					
Recruitment rate	# of subjects recruited/time	2 subjects/month/site	1 subject/m/site	N	Consider other recruitment strategies
Consent rate	% of subjects consenting	>20% acceptance	49% acceptance	Y	
Retention rate	% of subjects with complete data collection (T2, T3)	Complete T2 and T3 with ≥80% of subjects	T2 = 95% T3 = 87.5%	Y	
Perceived benefit	Post-intervention participant questionnaire	>85% will perceive benefit of WheelSeeU	100%	Y	
Resources (*n* = 4)					
Participant adherence					
WheelSeeU group	Complete 6 WheelSeeU sessions	>85% of subjects	95%	Y	
Control group	Complete 6 iWheel sessions	>85% of subjects	90%	Y	
Trainer adherence					
Peer-trainer	Recruit/retain peer-trainers	Attend 6 × 5 sessions	98%	Y	
Support-trainer	Recruit /retain support-trainers	Attend 6 × 5 sessions	98%	Y	
Data collection burden					
T1	Data collection time T1	>85% of subjects complete in ≤2 h	141 (36) min	N	Reduce the number of outcome measures collected
T2	Data collection time T1	>85% of subjects complete in ≤ 1.5 h	119 (43) min	N	Relax parameter for success
T3	Data collection time T1	>85% of subjects complete in ≤1.5 h	118 (56) min	N	
Translations	Translate and administer study materials in English and French	No issues	0 issues	Y	
Management (*n* = 4)					
Processing time	Time between initial subject contact to enrolment	Mean time is <10 days		N	Relax parameter for success
Combining data	Successfully combine data in English and French	No issues	0 issues	Y	
Study protocol administration	Study protocol checklist	Modifications can be made with minimal changes to protocol	Minimal change	Y	Allow for individual training if one subject drops out
Intervention fidelity	Observe and score peer-trainer and expert trainer administer the intervention	>90% on WheelSeeU Administrator Rating Form	90%	Y	
Safety (*n* = 1)					
Intervention	# of adverse events	No adverse events	0 adverse events	Y	
Data collection	# of adverse events	No adverse events	0 adverse events	Y	

#### Process indicators

*Recruitment rate*—It took 18 months to recruit 40 participants, at a rate of approximately one participant per month per site. However, recruitment rate was higher in Quebec (approximately two participants per month) compared to that in Vancouver (approximately two participants every 4 months).

*Consent rate*—A 49% consent rate was attained. Of the 120 participants contacted, 39 did not meet the inclusion criteria. Forty-one were not interested, and 40 gave their consent to participate in the study.

*Retention rate*—Forty individuals completed T1 assessments. The overall participant retention was 95% at T2 and 87.5% at T3. Of the 18 participants allocated to the experimental group, one was lost to follow-up at T2 and two more were lost to follow-up at T3. One participant withdrew from the study after two WheelSeeU sessions due to health reasons unrelated to the study and died shortly after. The other two participants were hospitalized after the completion of WheelSeeU for reasons unrelated to the study. Of the 22 participants allocated to the control group, one was lost to follow-up at T1 and one more was lost to follow-up at T3. One participant withdrew from the study after one iWheel session due to an unforeseen emotional issue associated with returning to the rehabilitation centre. The other participant died before completing T3 due to reasons unrelated to the study.

*Perceived benefit*—100% of participants who completed the WheelSeeU intervention felt that they received benefits for using a MWC from the WheelSeeU training program.

#### Resource indicators

*Participant adherence*—Participant adherence to the intervention was high in both groups. Not including participants who dropped out of the study (*n* = 5), 95% of participants allocated to the WheelSeeU intervention group and 90% of participants in the control group completed six of six training sessions. One participant in the intervention group completed five of six WheelSeeU sessions, while two participants in the control group completed five of six iWheel sessions. All others completed six of six sessions.

*Trainer recruitment and adherence*—A total of three peer-trainers were recruited and trained. The three peer-trainers (all males with spinal cord injury), who were 53.3 ± 10.0 years of age and had 20.3 ± 17.1 years of experiencing using a MWC, completed the WheelSeeU training. While an existing skill set was not a prerequisite for selecting peers, the peer-trainers in this study had various skills that may have influenced their competency for wheelchair training (e.g., athletic backgrounds, motivational speaking, coaching, teaching, and peer-mentorship experience). Three support-trainers (two occupational therapists, one kinesiologist), with 9.3 ± 1.5 years of clinical experience with MWC users, provided support to the peer-trainers. Of the 108 WheelSeeU sessions, one peer-trainer missed two sessions that were completed by the support-trainer.

*Participant and tester burden*—The burden of data collection was higher than anticipated, with mean (SD) testing times of 141 (36) min at T1, 119 (43) min at T2, and 118 (56) min at T3.

*Ability to translate/complete study in English and French*—There were no issues with translating or administering study materials in English or French.

#### Management indicators

*Participant processing time*—The mean (SD) subject processing time was 74 (80) days, which did not meet the criterion for success.

*Ability to combine data—*There were no issues with combining the English and French data.

*Administration of study protocol*—Successful administration of the study protocol was also achieved at both sites. Minor issues were addressed at monthly team meetings, such as how to proceed if a participant or trainer missed a training session. In both cases, the study proceeded with one participant and one trainer. A change to the protocol was made in one circumstance when one participant in the control group dropped out of the study before completion of the control group didactic sessions. It was decided to complete the sessions with the control group trainer and one participant only.

*Fidelity*—The WheelSeeU Administration Rating form was completed by research coordinators at both sites on six separate occasions. A mean (SD) score of 90 (5)% revealed that fidelity of the WheelSeeU intervention was attained. One issue that was raised at both sites was that the support-trainer took more of a lead role than intended during the first few WheelSeeU sessions. As the peer-trainer gained experience and felt more comfortable and confident in their role, they began to take on the lead role as was intended.

#### Safety

*Intervention*—The WheelSeeU protocol was *safe* for participants, as there were no adverse events in delivering the WheelSeeU intervention.

*Data collection*—There were no adverse events during data collection.

There was one adverse incident with the peer-trainer during the WheelSeeU intervention, which did not result in injury or serious concern. The peer-trainer tipped backwards in his wheelchair during demonstration of one of the skills and used the opportunity to teach learners about safe falling techniques.

## Discussion

The results of this study confirm that the peer-led WheelSeeU study protocol was feasible to administer to community-living older adults. Following minor modifications to address three feasibility issues (recruitment rate, burden of data collection, and subject processing time), findings support conducting a larger clinical trial to evaluate the effectiveness of peer-led WheelSeeU training for older adults in the community.

### Process

Further consideration of how to best target older community-living adults who use MWCs may improve feasibility for recruiting older adults MWC users. About 6 months into recruiting, the eligibility criteria were slightly revised from ≥55 to ≥50 years of age to increase the potential participant pool. Revising eligibility criteria without reducing scientific rigor has been suggested as one approach for recruiting wheelchair users [35]. Although the WheelSeeU study used various creative recruitment strategies at both sites to target wheelchair users, as suggested by Nary et al. [35], recruitment posed a challenging issue. Recruitment methods in Vancouver included clinicians and vendors describing the study to inpatients and outpatients at a local rehabilitation centre, posters, and presentations to special interest groups in the community, and mail-outs to potential participants as identified by hospital records. Although we tried to reach additional participants through wheelchair vendors in the community, there was some reluctance to ‘push’ a product that was not being sold by the company. All 26 participants in Québec City were identified and recruited by a clinician through a database of wheelchair users at the Institut de réadaptation en déficience physique de Québec, which may explain why recruitment rates were higher in Québec City compared to Vancouver. More research is needed to determine best methods for recruiting older community-living wheelchair users.

Although a 49% consent rate is considered very high in rehabilitation research, recruitment issues identified in this study suggest that recruiting participants during initial rehabilitation may not be the best time for intermediate or advanced MWC training. In addition to clinician-perceived barriers of conducting wheelchair skills training during rehabilitation [12], issues of competing priorities, physical and psychological adjustments to mobility impairment, and lack of real-world experiences with using a wheelchair may influence the likelihood of new MWC users to participate. Therefore, community-based training programs like WheelSeeU may be better suited for older adults after they have had some time to experience the task demands of MWC use in the community.

It is promising that participant retention was high at both time points (95% at T2 and 87.5% at T3) in both the experimental and control groups. All participants who completed the WheelSeeU intervention felt they benefited from the training, which has prompted the design of a qualitative study to obtain richer data about participants’ experiences and perceptions with a peer-led MWC training program. The WheelSeeU study used an active control group (iWheel) in efforts to increase participant adherence, which seemed to be well received by participants. While having an active control group may have been a less pragmatic approach in the evaluation of a novel intervention, this approach minimized threats to internal validity by controlling for researcher attention and travel to sessions [36].

### Resources

The burden of data collection was higher than anticipated; however, no participants indicated issues with testing times. In fact, comments were made about enjoying the time with the tester in many cases. Participant and tester burden may be reduced through elimination of the objective WST from the protocol, which takes approximately 30 min to complete. Although WST-Q scores are highly correlated with objective WST scores [28], the WST-Q may be a more valid indication of wheelchair skills capacity because participants are not penalized for technical testing errors (e.g., touching the line). Additionally, collecting only the participation component of the LLFDI may further reduce testing by 15–20 min. Since WheelSeeU targets improved MWC mobility for the purpose of increasing participation outcomes, future trials should consider patient-reported outcomes to fully understand the impact of WheelSeeU [37].

It is promising that both participant and peer-trainer adherence to the intervention was high. Although all participants stated perceived benefits of WheelSeeU, the exact reasons for adherence are unknown. Qualitative interviews with participants who completed WheelSeeU may provide considerable insight about perceived facilitators and barriers influencing adherence and the important characteristics of peer-trainers (e.g., age, gender, previous experiences) that may inform the development of future studies and sustainable community-based programs. Qualitative interviews with participants in the control group may have provided useful information about the “attention-control” activity. The iWheel sessions provided participants with social activity and with resources about their community, which may have had potential perceived benefits for participants. Finally, qualitative interviews with those who accept and decline to participate in intervention research may also provide a better understanding of how to recruit MWC users for community-based programs. Findings from this study also support the feasibility of recruiting and maintaining a peer-trainer, which may positively contribute to the sustainability of community-based wheelchair training programs in the future.

It is plausible that peer-led MWC training may be delivered to larger groups of MWC users in the community without the need for clinician presence, thus maximizing resources and potentially decreasing costs. However, prior to this, it is prudent to ensure that peer-trainers have the necessary characteristics and skill sets. This feasibility study allowed us to gain insight on some of the important characteristics of a peer-trainer (e.g., previous coaching/ peer-mentorship/teaching experience, wheelchair skills, wheelchair use confidence), thus suggesting prerequisites for future recruitment of peer-trainers. With the right peer-trainers, it is possible that clinicians or MWC training experts could be consulted as required. The feasibility nature of this study allowed us to explore the important characteristics of peer-trainers. However, due to pragmatic reasons (i.e., slow recruitment) and to maximize safety, the trainer to participant ratio was 2:2.

### Management

While success on subject processing time was not achieved, the fact that participants remained interested in the study after a period of approximately 3 months is promising. For participants who were contacted during a rehabilitation stay, regular contact was maintained between the initial screening and enrolment [35]. This also highlights the importance of providing wheelchair training at times that are convenient for participants and supports the necessity for wheelchair training that continues after discharge from initial rehabilitation.

Intervention fidelity is critical to knowledge translation of evidence-based programs. With ~15 h of training, older MWC users and clinicians were able to administer a self-efficacy enhanced wheelchair training program. However, in some cases, the support-trainer took more of a lead role in the training than was intended. As the peer-trainer gained experience and felt more comfortable and confident in their role, they began to take to lead as intended. As stated by Green [38], this reinforces the importance of peer-selection and ensuring peers receive adequate training. Although the current WheelSeeU program was co-led by a peer-trainer and a support-trainer, the future intent of WheelSeeU is to provide a peer-led program without the need for a healthcare professional. Future studies of peer-led wheelchair training should also consider ecological validity and the inability to control all protocol parameters in a real-world application of WheelSeeU. It has been suggested that tradeoffs between maintaining experimental control and fidelity of evidence-based programs maximizing fit to the new context should be considered in research among individuals with disabilities [39].

### Safety

Findings from this study confirm that a wheelchair training program administered by a peer and a health care professional is safe. However, as previously mentioned, the future intent of WheelSeeU is to be solely administered in the community by a peer-trainer. Recent findings from focus groups conducted during the development of a peer-led program highlight some concern for programs led solely by lay peers [40], but there is evidence that supports competencies of peer-trainers when adequate training is provided [41]. Moreover, community-living MWC users should be given the autonomy and choice to make decisions about how they participate in community-based programs.

### Limitations

The consent rate (49%) was based on the number of individuals contacted by research investigators during the study period. This number may be underestimated, as there is no way to track the number of people who may have been contacted through snowball methods or by world-of-mouth from clinicians who were not a part of the study team.

There may have been pragmatic issues with slow enrollment and an intervention designed for dyadic training. In order to perform the most robust randomization procedures (i.e., group allocation upon completion of baseline measures), while reducing the chances of dropout due to long wait periods between baseline testing and start of the intervention period, randomization was done in blocks of two. This means that as two participants were enrolled, they were allocated in pairs to either the intervention or the control group. Restricting training to pairs of participants was recognized as a limitation in this study. Future studies may consider one-on-one training or training in small groups.

While assessing peer-trainer adherence was an a priori objective, we did not include support-trainer or iWheel trainer adherence as a study objective. Both the support-trainer and iWheel trainer were part of the extended research team and maintained regular contact with the study investigators, including attending bimonthly team meetings to ensure any concerns could be addressed as they arose. Details about support-trainer and iWheel trainer adherence may have provided useful information pertaining to this study and should be considered for future feasibility studies.

Finally, details about peer-trainer training and intervention fidelity may have strengthened the results of this study. Implementation of a test at the end of the 2-day training session may help to ensure the peer-trainer and support-trainer are prepared to administer the intervention. However, study investigator (KB) has more than 10 years of experience in wheelchair skills training and thus should be able to attest to trainer readiness. Additionally, the WheelSeeU Administrator Rating form could have been administered more frequently to understand fully how WheelSeeU was delivered and whether it was administered as intended. Specifically, more information on how the lead roles shifted from the support-trainer to the peer-trainer would provide more insight into factors that may be taken into consideration when training the peer-trainer. It is possible that an additional day of training would have been useful for practice. It is possible that WheelSeeU sessions could be videotaped; however, it was felt that this could hinder organic discussions.

## Conclusions

Self-efficacy enhanced peer-led wheelchair training provides a feasible option that may contribute to the development of a needed community-based wheelchair training continuum for older wheelchair users. The present study demonstrates that with the support of a clinician, it is feasible for peers to teach older MWC users about using their wheelchair. Further examination of the efficacy of community-based peer-led programs in a larger randomized controlled trial is warranted.

## Abbreviations

iWheel: Information for using a wheelchair
MWC: Manual wheelchair
RCT: Randomized controlled trial
SD: Standard deviation
WheelSeeU: Wheelchair training Self-Efficacy Enhanced for Use
